# Electrospun PVA Polymer Embedded with Ceria Nanoparticles as Silicon Solar Cells Rear Surface Coaters for Efficiency Improvement

**DOI:** 10.3390/polym10060609

**Published:** 2018-06-04

**Authors:** Effat Samir, Mohamed Salah, Ali Hajjiah, Nader Shehata, Marwa Fathy, Aya Hamed

**Affiliations:** 1 Center of Smart Nanotechnology and Photonics (CSNP), SmartCI Research Center, Alexandria University, Alexandria 21544, Egypt; effat_samir@mena.vt.edu (E.S.); m.salah@mena.vt.edu (M.S.); aya_ali@mena.vt.edu (A.H.); 2 Department of Electrical Engineering, Faculty of Engineering, Alexandria University, Alexandria 21544, Egypt; 3 Department of Engineering Mathematics and Physics, Faculty of Engineering, Alexandria University, Alexandria 21544, Egypt; 4 Kuwait College of Science and Technology, Doha Area, Safat 13133, Kuwait; 5 Electrical Engineering Department, College of Engineering and Petroleum, Kuwait University, Kuwait City 13133, Kuwait; ali.hajjiah@ku.edu.kw; 6 USTAR Bio-Innovation Center, Utah State University, Logan, UT 84341, USA; 7 The Bradley Department of Electrical and Computer Engineering, Virginia Tech, Blacksburg, VA 24061, USA; 8 Electronic Materials Department, Advanced Technology and New Materials Research Institute (ATNMRI), City of Scientific Research and Technology Applications (SRTA-City), New Borg El-Arab City, Alexandria 21934, Egypt; naashehata@yahoo.com

**Keywords:** solar cells, coaters, electrospun, ceria nanoparticles, fluorescence

## Abstract

This paper introduces electrospun nanofibers embedded with ceria nanoparticles as silicon solar cells coaters, showing their influence on the solar cells efficiency. Ceria nanoparticles can be synthesized to have formed oxygen vacancies (O-vacancies), which are associated with converting cerium ions from the Ce^4+^ state ions to the Ce^3+^ ones. These O-vacancies follow the rule of improving silicon solar cellconductivity through the hopping mechanism. Besides, under violet excitation, the reduced trivalent cerium Ce^3+^ ions are directly responsible for down-converting the un-absorbed violet or ultra-violet (UV) wavelengths to a resulted green fluorescence emission at ~520 nm. These are absorbed through the silicon solar cells active layer. When electrospun Poly(vinyl alcohol) (PVA) is embedded with ceria nanoparticles on the rear surface of silicon solar cell, a promising enhancement in the behavior of solar cells current–voltage (I–V) curve is observed. The efficiency has improved by about 24% of its initial value due to the mutual impact of improving both electrical conductivity and optical conversions from the higher surface-to-volume ratio of electrospun nanofibers embedded by ceria nanoparticles. The solar cell efficiency improvement is due to the mutual impact of both optical down-conversion and better electric paths via the used nanocomposite. The added nanostructures coating can utilize part of the transmitted UV or violet spectrum through the cell as optical conversion from violet to the visible region. In addition, the formed active tri-valent states are associated with O-vacancies which can help in a better conductivity of the generated photoelectrons from the cell through the hopping mechanism. The PVA nanofibers host offers a better distribution of ceria nanoparticles and better conductivity paths for the photoelectrons based on the better surface-to-volume ratio of the nanofibers.

## 1. Introduction

**Figure 1:**
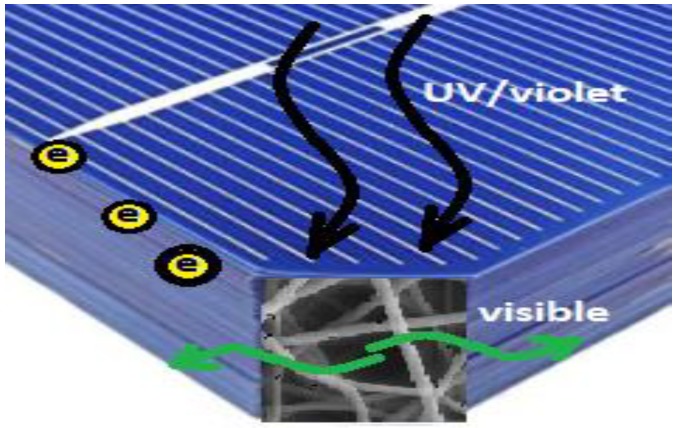
Simple schematic diagram to show the idea of the work; the nanocomposite rear coater is converting the transmitted UV/violet to visible light. In addition, the nanocomposite helps to offer better electric paths for the generated photoelectrons to the electrodes.

Silicon (Si) is the most dominant commercial material used in the solar cells industry [1]. Although Si accounts for about 90% of the worldwide solar cells production, it still suffers from many issues that lead to an overall efficiency reduction [2,3]. However, these issues can be categorized into two main types: optical and electrical losses. Consequently, many researchers tend to enhance Si solar cells performance in order to reach the maximum possible efficiency from such a dominant renewable energy. Transforming the un-absorbed sun radiation photons energy to photons with energies that can be utilized by the solar cell could significantly enhance the efficiency of the solar cell [4,5]. Optical conversion processes are considered to be the best ways to improve optically the Si solar cells efficiency by exploiting the un-absorbed infra-red (IR) or ultra-violate (UV) solar spectra through optical conversions or photoluminescence mechanisms [6]. Meanwhile, electrical losses are another source of problems which can be shown up from the metal coverage shadowing losses, Ohmic losses, temperature, and metal-insulator-semiconductor (MIS) barriers [7]. These electrical issues mainly affect the cell conductivity and electrical I–V curve including open circuit voltage (V_OC_) and short circuit current (I_SC_), therefore reducing the overall power conversion efficiency (PCE) of the solar cell. Coating solar cells or panels with nanostructures have recently been investigated in extensive research to enhance the overall efficiency of the cells [8]. In this paper, the aim is to coat the rear surface of silicon (Si) cell with a thin layer of optical conductive electrospun nanofiber mat to improve the cell PCE. Cerium oxide (ceria) nanoparticles embedded in electrospun nanofibers, has been investigated as Si solar cells backside coaters to improve Si solar cell efficiency. Ceria nanoparticles attract a great research and commercial interests due to its capacity of oxygen storage and optical characteristics of optical conversions in many applications related to medical, environmental, and sustainable energy fields [9,10]. Ceria nanoparticles can be synthesized to be active with tri-valent cerium ions associated with charged oxygen vacancies [11]. These Ce^3+^ ions associated with the oxygen vacancies formations play the role of making ceria nanoparticles being a conductive material using hopping mechanism. Besides, Ce^3+^ ions are responsible for a visible fluorescence emission, under violet or near UV-excitation photons. Therefore, the active tri-valent states of cerium ions inside ceria nanoparticles can beneficial for different applications based on both electrical and optical characteristics. In this paper, we have electrospun the active ceria nanoparticles within poly (vinyl alcohol) (PVA) nanofibers host, and then exploited the main optical and electrical features of ceria to improve the efficiency of solar cells as backside rear coaters. Electrospun nanofibers host here is beneficial for ceria nanoparticles to offer a solid host of the nanoparticles with higher surface to volume ratio. In this work, the embedded ceria nanoparticles, synthesized by chemical precipitation, with PVA solution are mixed together as the solution feeder in the electrospinning process to generate the final nanofibers and deposit it directly on the Si solar cells backside surface. During the experimental work, different electrical and optical characterization measurements such as conductivity, fluorescence emission intensity, absorbance, and the allowed bandgap investigated for the electrospun nanocomposite. These characterization was obtained to proof how active are ceria nanoparticles after the electrospinning process. The activity of ceria nanoparticles determines the existence of the formed charged O-vacancies which are responsible for the mutual impact of improving both electric conductivity and optical down-conversions of Si solar cells. Then, the solar cells are characterized whether in both cases uncoated and coated cells, to prove the efficiency improvement with the added layer of nanocomposite. This is based on mutual contribution of optical down-conversion of violet to visible emission due to the tri-valent cerium ions. In addition, the tri-valent cerium ions have associated O-vacancies which can be better electric paths for generated photoelectrons. The nanofibers host offers better surface-to-volume ratio for a better distributing cerium ions. Figure 1 shows a schematic diagram about the concept of mutual contribution of both better photoelectrons paths and optical down-conversion from UV/violet to visible emission.

## 2. Materials and Methods

**Figure 2:**
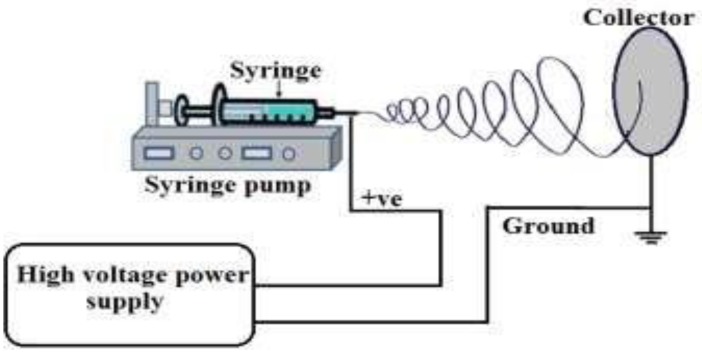
Used electrospinning setup schematic diagram.

Chemical precipitation is selected as the synthesis procedure of ceria nanoparticles for its simplicity and cheap used chemicals [12]. In this procedure, each sample is prepared for 40 mL of distilled water contains 0.5 g of cerium (III) chloride heptahydrate (99.9%, Sigma-Aldrich, St. Louis, MO, USA) and 1.6 mL of ammonium hydroxide of 1.6 mL as a catalyst. This solution is stirred in 50 °C water bath for 2 h. Then, the solvent is stirred at room temperature overnight. In parallel, PVA solution is formed by mixing 10 g of commercial PVA pellets in 90 mL of distilled water. This weight concentration; of 10 wt %, is selected based on electrospinning trials of PVA nanofibers which is the optimum concentration to form fibers with nearly no beads. PVA solution is heated at 100 °C for 30 min, and then overnight stirred. Then, ceria nanoparticles are added to the prepared PVA polymer, and then the mixture is stirred for 30 min before it is entering the electrospinning process. The Electrospinning setup consists of high voltage power supply (Spellman High Voltage Electronics corporation model CZE1000R, Hauppauge, NY, USA) to form high potential difference between both feeder and collector, a syringe pump (NE1000Single Syringe Pump, New Era, Farmingdale, NY, USA) to pump the nanocomposite solution which is inserted in a syringe with a metallic needle as a feeder of the solution, and a metallic collector as a target. A schematic of the used Electrospinning setup is shown in Figure 2. The distance between the needle tip and the collector is fixed at 15 cm, with voltage difference of 25 kV. The flow rate of the polymer solution is 2 mL/h for 30 min running time per sample. For coating the solar cells, each polycrystalline cell (of dimensions 2 in. × 2 in. and thickness of 200 micron, PG instruments, Lutterworth, England, UK) is fixed at the collector of the electrospinning setup in such a way that the rear side of the cell is exposed to the deposited nanofibers.

The electrospun PVA polymer embedded with the synthesized ceria nanoparticles are optically characterized by measuring its optical absorbance, which leads to bandgap calculations as will be shown later, and fluorescence intensity curves. Optical characterization measurements are obtained by UV–Vis–NIR spectrometer (PG 92 spectroscopy, Lutterworth, England, UK), which is used to detect the absorbance as a function of optical spectrum of wavelength range between 300–800 nm. Then, in order to measure the photoluminescence intensity, the experimental apparatus used is the same setup discussed in other of our related research papers such as Ref. [11]. In this setup, violet LED of a center wavelength of 430nm (Thorlab, Newton, NJ, USA) as optical excitation source is exposed to the synthesized nanoparticles solution and a monochromator is located beyond the excited sample perpendicular to the excitation source for minimum scattering impact. Then, the optical signal which passed by the monochromator is detected by photomultiplier tube (PMT, Newport 77360, Irvine, CA, USA) followed by a power meter (Newport, 1918R, Irvine, CA, USA). The power meter monitors the optical visible fluorescent emission. The mean synthesized nanoparticle size was observed by transmission electron microscopy (TEM, JEOL, Peabody, MA, USA), with accelerating potential of 80KV. Surface morphology and diameter size of electrospun nanofibers were investigated using scanning electron microscopy (FEI Quanta 200, Graz, Switzerland). Coated solar cells are characterized by Keithley sourcemeter of model (2450C) (Tektronix, Beaverton, OR, USA) with illumination source of Oriel Xenon lamp (Irvine, CA, USA).

## 3. Results& Discussion

### 3.1. Optical Characterization of the Electrospun Nanocomposite Nanofibers

**Figure 3:**
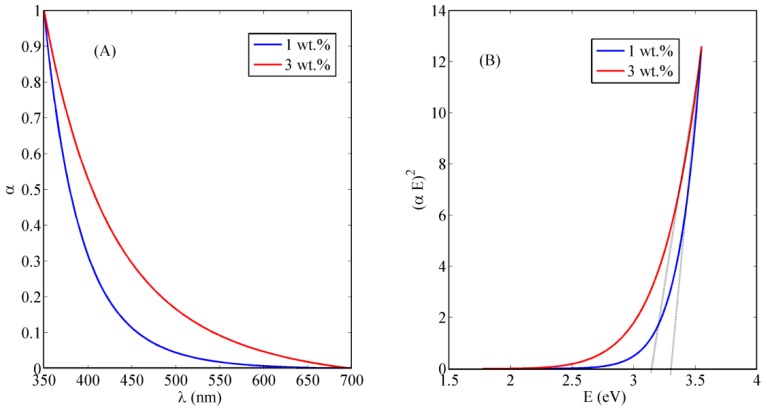
(**A**) Absorbance dispersion and (**B**) direct allowed bandgap calculations of polyvinyl alcohol (PVA) nanofibers with in-situ embedded different weight concentrations of ceria nanoparticles (NPs).

**Figure 4:**
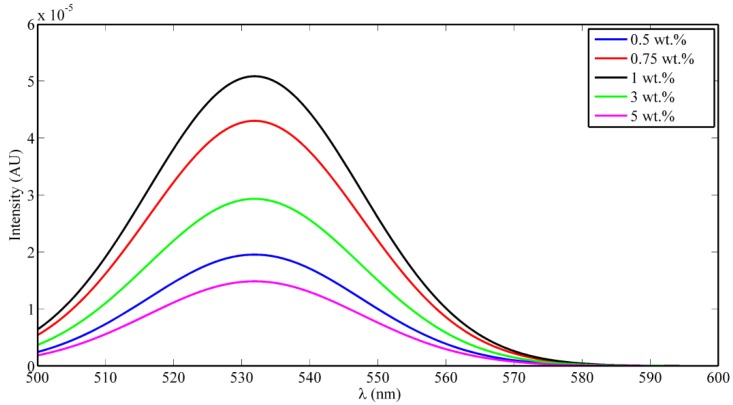
Fluorescence intensity PVA nanofibers (NFs) with in-situ embedded different concentrations ceria NPs.

**Figure 5:**
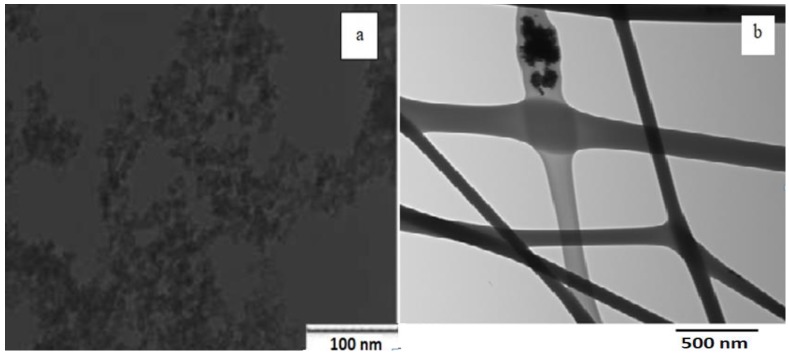
(**a**) Transmission electron microscope (TEM) of ceria NPs and (**b**) Scanning Electron Microscope (SEM) of nanofibers with embedded ceria with the arrows show the agglomerated parts of ceria NPs over the PVA nanofibers.

The absorbance dispersion of the synthesized nanocomposite is measured and shown in Figure 3A. It can be observed that the absorbance starts to increase around the wavelength of 400 nm, indicating the optical absorbance of the tri-valent cerium ions which should be around 3 eV of trap state inside the non-stoichiometric state of CeO_2−*x*_. From the absorbance dispersion, the direct allowed bandgap can be calculated from the relation between (*αE*)^2^ and *E*. Figure 3B shows the relation between (*αE*)^2^ versus *E*, and the intersection of the linear extension of the curve with *E*-axis represents the value of bandgap [13]. The resulted allowed bandgap range of range down to 3.5 eV is matching with other literatures, which confirm the formation of active ceria with some quantity of tri-valent cerium ions and corresponding O-vacancies [14]. To confirm the optical activity of embedded ceria inside the electrospun nanofibers, the fluorescence emission is detected for the synthesized nanocomposite under 430 nm excitation. Fluorescence emission appears at wavelength approximately equals to 520 nm due to the active ceria nanoparticles which has fluorescence emission according to the tri-valent cerium ions. In the range of <1 wt % of ceria NPs, as shown in Figure 4, the fluorescence intensity peak increases with increasing the concentration due to more formed optical tri-valent states of cerium ions with corresponding electron transitions of 5d–4f levels [11]. However, the increase of higher ceria nanoparticles concentration above 1 wt % leads to decrease fluorescence emission intensity peaks. This may be due to static fluorescence quenching effect, which can dominantly appear at higher ceria nanoparticles concentrations greater than 1 wt %, leading to this decrease in fluorescence emission. Transmission electron microscope (TEM) image of ceria nanoparticles and Scanning Electron Microscope (SEM) image of PVA nanofibers with embedded ceria nanoparticles in-situ are shown in Figure 5a,b. The average grain size of ceria nanoparticles is ~6 nm and the formed nanofibers’ mean diameter is ~150 nm. From Figure 5b, it can be shown that some ceria nanoparticles are agglomerated on the electrospun nanofibers. However, it can not be used some surfactant to separate such agglomeration, to avoid any impact on the oxygen vacancies and keeping the active tri-valent cerium ions inside the nanofibers.

### 3.2. Electrospun the Nanocomposite onto the Rear Side of Silicon Solar Cells

**Figure 6:**
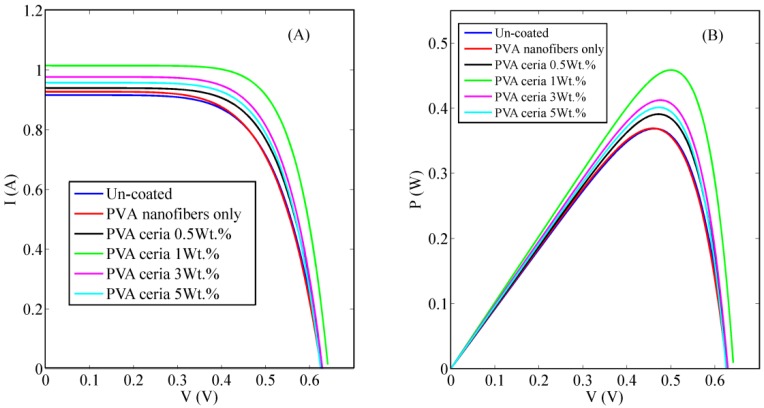
Comparison between uncoated and different concentrations of electrospun ceria nanoparticles embedded in PVA nanofibers coating solar cells (**A**) I–V curve; (**B**) P–V curve.

Figure 6 shows the influence of introducing different concentrations of the ceria nanoparticles embedded in the electrospun PVA nanofibers upon Si solar cells I–V and P–V curves, respectively. A detailed comparison between uncoated and coated Si solar cells electrical parameters has presented in Table 1, which shows a promising improvement in the overall efficiency and some other solar cells electrical parameters. It is obvious that coating the rear surface of Si solar cells with 1 wt % concentrations of ceria nanoparticles embedded in PVA nanofibers shows the highest power conversion efficiency (PEC) improvement among the rest of concentrations, which was also the highest obtained intensity in the fluorescence emission spectra curve. The obtained improvement was from 14.74% to 18.39% that is corresponding to about 24% increase from its’ initial value. The calculated electrical parameters obtained from the measured I–V curves, shows the improvement of the short circuit current (I_SC_) due to the effect of the synthesized nanoparticle coating studied features, besides a quite increase within open circuit voltage (V_OC_) and fill factor (FF). This can be explained by the mutual effect of both optical conversion of ceria nanoparticles along with its electric conductivity. Generally, UV and violet spectra are not absorbed by silicon solar cells, so our added nanostructures coating can utilize the unabsorbed spectrum of the cell in a better way through optical conversion from violet to visible region. As cerium ions in tri-valent ionization states can convert violet to visible emission based on 5d–4f transition [14]. In addition, the formed active tri-valent states are associated with O-vacancies which can help in a better conductivity of the generated photoelectrons from the cell through hopping mechanism. The PVA nanofibers host offers a better distribution of ceria nanoparticles and better conductivity paths for the photoelectrons based on the better surface-to-volume ratio of the nanofibers. Then, it is a mutual impact of both optical down-conversion and better electric paths via the used nanocomposite. Our results show that 1 wt % of ceria nanoparticles concentration inside the fibers gives the highest efficiency. Beyond this concentration, the efficiency starts to decrease due to possible scattering and optical quenching effects by the higher levels of ceria concentrations.

## 4. Conclusions

This paper introduces a promising study of ceria nanoparticles embedded in PVA nanofibers as a backside coating layer on silicon solar cells. The presented work shows optical characterization of the synthesized nanoparticles after embedding it in the electrospun PVA polymer nanofibers. The experimental results show that ceria nanoparticles are still active inside the electrospun nanofibers mat. The obtained visible fluorescence emitted under violet excitation, besides the results of the band gap, confirm the formation of Ce^3+^ trap states which are associated with the formation of charged oxygen vacancies. These oxygen vacancies could increase the conductivity for electrons in the host nanoparticles through hopping mechanisms. After electrospinning, the nanocomposite of ceria nanoparticles inside the PVA polymer, a promising improvement in the solar cell efficiency has been observed due to the mutual impact of improved electric conductivity and optical down-conversion mechanisms. The solar cells efficiency improvement was about 24% from its initial value after electrospinning 1 wt % ceria nanoparticles concentration inside PVA nanofibers on the solar cells rear side surface based on mutual electrical and optical conversion properties.

## Figures and Tables

**Table 1:** Comparison of electrical parameters between uncoated and coated solar cell with electrospun PVA embedded with ceria nanoparticles.

Condition	Concentration (mg/mL)	V_OC_(V)	I_SC_ (A)	F.F.	J_SC_ (A/m^2^)	Efficiency (η%)
Uncoated Solar cell	Uncoated	0.6320	0.9165	0.6620	366.6	14.74
Nanocomposite coated solar cell	PVA only	0.6282	0.9279	0.6313	371.2	14.75
	0.5 wt %	0.6308	0.9400	0.6667	376.0	15.63
	1 wt %	0.6414	1.0153	0.7079	406.1	18.34
	3 wt %	0.6320	0.9774	0.6760	390.9	16.49
	5 wt %	0.6270	0.9581	0.6792	383.2	16.04
